# Supplementary material for: Cocaine-induced endocannabinoid signaling mediated by sigma-1 receptors and extracellular vesicle secretion
Source: eLife. 2019 Oct 9;8:e47209. doi: 10.7554/eLife.47209 (PMC6850780; doi:10.7554/eLife.47209)
Supplement: Transparent reporting form [file elife-47209-transrepform.docx]

***eLife’s* transparent reporting form**

We encourage authors to provide detailed information *within their submission* to facilitate the interpretation and replication of experiments. Authors can upload supporting documentation to indicate the use of appropriate reporting guidelines for health-related research (see [EQUATOR Network](http://www.equator-network.org/%20)), life science research (see the [BioSharing Information Resource](https://biosharing.org/)), or the [ARRIVE guidelines](http://www.plosbiology.org/article/info:doi/10.1371/journal.pbio.1000412) for reporting work involving animal research. Where applicable, authors should refer to any relevant reporting standards documents in this form.

If you have any questions, please consult our Journal Policies and/or contact us: [editorial@elifesciences.org](mailto:editorial@elifesciences.org).

**Sample-size estimation**

- You should state whether an appropriate sample size was computed when the study was being designed
- You should state the statistical method of sample size computation and any required assumptions
- If no explicit power analysis was used, you should describe how you decided what sample (replicate) size (number) to use

The experiments were designed using estimates of effect size and standard error derived from prior experience (pilot experiments) with techniques described in Materials and Methods. These values were then used in power analysis calculations, using the program G-Power (version 3.1.9.4, University of Dusseldorf, Germany, <http://www.gpower.hhu.de/en.html>) to determine sample sizes.

This information is included in the Materials and Methods section of the manuscript.

Please outline where this information can be found within the submission (e.g., sections or figure legends), or explain why this information doesn’t apply to your submission:

**Replicates**

- You should report how often each experiment was performed
- You should include a definition of biological versus technical replication
- The data obtained should be provided and sufficient information should be provided to indicate the number of independent biological and/or technical replicates
- If you encountered any outliers, you should describe how these were handled
- Criteria for exclusion/inclusion of data should be clearly stated
- High-throughput sequence data should be uploaded before submission, with a private link for reviewers provided (these are available from both GEO and ArrayExpress)

Please outline where this information can be found within the submission (e.g., sections or figure legends), or explain why this information doesn’t apply to your submission:

The numbers of replicates for each experiment are shown in the figures themselves, when bar graphs are used, or reported in the figure captions. Outliers were not identified by any statistical criteria, and therefore, if present, were included in datasets used in the analyses.

**Statistical reporting**

- Statistical analysis methods should be described and justified
- Raw data should be presented in figures whenever informative to do so (typically when N per group is less than 10)
- For each experiment, you should identify the statistical tests used, exact values of N, definitions of center, methods of multiple test correction, and dispersion and precision measures (e.g., mean, median, SD, SEM, confidence intervals; and, for the major substantive results, a measure of effect size (e.g., Pearson's r, Cohen's d)
- Report exact p-values wherever possible alongside the summary statistics and 95% confidence intervals. These should be reported for all key questions and not only when the p-value is less than 0.05.

Please outline where this information can be found within the submission (e.g., sections or figure legends), or explain why this information doesn’t apply to your submission:

Means ± s.e.m. are used throughout to report measures of centricity and dispersion. A table describing means and 95 % confidence intervals for each experiment is included with this report. Statistical tests were determined by the number of groups and treatments to be compared. An omnibus test was when necessary statistical assumptions could be met. Thus, in experiments where repeated measures could be obtained from the same subjects, samples, or cells (e.g. time course data), a repeated-measures ANOVA test was used. When repeated measures were not performed, and group size was >2, a one-way ANOVA was used. Post-hoc analyses (Tukey’s, Dunnett’s, or Bonferroni’s multiple comparison tests) were determined by the type of omnibus test, as well as the nature of the multiple comparisons (pairwise rows and columns, comparison to control columns, main effects versus interactions). When only 2 groups of data were compared, a Student’s t-test was used. In all cases a two-tailed p value of 0.05 was considered the minimum for significance. Actual p values are reported for all omnibus tests, unless p < 0.0001, and the statistical information is reported in the figure captions. In immunoprecipitation experiments, co-localization was determined from observed association on Western blots, and therefore, statistical tests were not used (Fig. 1F, 1G; Fig. 3B, 3D; Fig. 5C; Fig. S2C).

This information is reported in the **Quantification, Statistical Analysis and Reporting**

section of the manuscript.

(For large datasets, or papers with a very large number of statistical tests, you may upload a single table file with tests, Ns, etc., with reference to sections in the manuscript.)

**Group allocation**

- Indicate how samples were allocated into experimental groups (in the case of clinical studies, please specify allocation to treatment method); if randomization was used, please also state if restricted randomization was applied
- Indicate if masking was used during group allocation, data collection and/or data analysis

Please outline where this information can be found within the submission (e.g., sections or figure legends), or explain why this information doesn’t apply to your submission:

Group membership was determined by genotype where transgenic mice were used. In in vitro electrophysiology studies, recordings from untreated control brain slices were interleaved with recordings from brain slices from the same animal, in which drug pre-incubation had occurred. In cell biology experiments, mice were chosen for experiments depending upon date of arrival from the supplier. In this way, mice were assigned to groups according availability and to the experimental procedures to be performed that day. In most cases, brain tissue from each mouse was used in both control and treatment conditions. NG-108 cell culture dishes were selected randomly from those available in the tissue incubator.

This information is in the Group allocation section of Materials and Methods

**Additional data files (“source data”)**

- We encourage you to upload relevant additional data files, such as numerical data that are represented as a graph in a figure, or as a summary table
- Where provided, these should be in the most useful format, and they can be uploaded as “Source data” files linked to a main figure or table
- Include model definition files including the full list of parameters used
- Include code used for data analysis (e.g., R, MatLab)
- Avoid stating that data files are “available upon request”

Please indicate the figures or tables for which source data files have been provided:

A single Microsoft Excel file containing, statistical tests, degrees of freedom, p values, means and 95 % confidence intervals, for each experiment, as well as the location of this information in the manuscript, has been provided.
